# Supplementary material for: Identification of photoperiod-induced specific miRNAs in the adrenal glands of Sunite sheep (Ovis aries)
Source: Front Vet Sci. 2022 Jul 22;9:888207. doi: 10.3389/fvets.2022.888207 (PMC9354845; doi:10.3389/fvets.2022.888207)
Supplement: Supplementary file 1 [file Data_Sheet_1.ZIP › Supplementary file/miRDeep2 file.zip!/miRDeep2 file/Novel_Structure/SPLP42A3/Novel_330.pdf]

Provisional ID : NC_019469.2_21929
Score total : 0.1
Score for star read(s) : -1.3
Score for read counts : 0
Score for mfe : -0.1
Score for randfold : 1.6
Score for cons. seed :
Total read count : 817
Mature read count : 817
Loop read count : 0
Star read count : 0

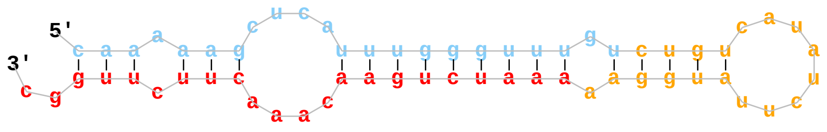

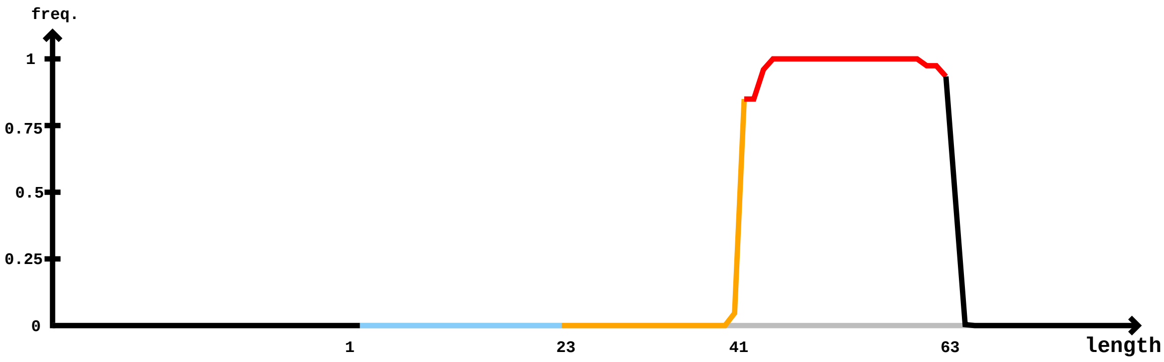

Star Mature

```
5'- cuggacaaaaaaagauuauauugaguugguCaaaaagcucauuuggguuuguCugucauaucuuauggaaAaaucugaacaaacuucuuggcCaauccagugugaaacaca   -3'    exp
    ...............((((((((.(((((((((.(((....(((((((((.(((((........))))).)))))))))....))).))))))))).))))))))......     reads     mm     sample
    .....................................................................aaaaucugaacaaacuuUuu......................     18        1      seq
    .....................................................................aaaaucugaacaaacuuUuugg....................     19        1      seq
    .....................................................................aaaaucugaacaaacuucuuggc...................     1         0      seq
    ......................................................................aaaucugaacaaacuuUuu......................     3         1      seq
    ......................................................................aaaucugaacaaacuuUuugg....................     14        1      seq
    ......................................................................aaaucugaacaaacuuUuuggc...................     637       1      seq
    ......................................................................aaaucugaacaaacuucuuggc...................     2         0      seq
    ........................................................................aucugaacaaacuuUuuggc...................     16        1      seq
    ........................................................................aucugaacaaacuucuuggc...................     1         0      seq
    ........................................................................aucugaacaaacuuUuuggcc..................     73        1      seq
    .........................................................................ucugaacaaacuuUuuggcc..................     30        1      seq
    .........................................................................ucugaacaaacuuUuuggcca.................     3         1      seq
```
